# An Outbreak Preparedness and Mitigation Approach in Home Health and Personal Home Care During the COVID-19 Pandemic

**DOI:** 10.1177/1084822320933567

**Published:** 2020-11

**Authors:** William R. Mills, Susan Sender, Karen Reynolds, Joseph Lichtefeld, Nicholas Romano, Melissa Price, Jennifer Phipps, Leigh White, Shauen Howard, Rexanne Domico

**Affiliations:** 1 BrightSpring Health Services, Louisville, KY, USA

**Keywords:** COVID-19, home health care, outbreak, Coronavirus, home care, personal care

## Abstract

The acute respiratory disease COVID-19, caused by the novel Coronavirus SARS-CoV-2, is a worldwide pandemic affecting millions of people. The methodology that organizations who provide home health and personal home care services are using to respond to this pandemic has not yet been characterized. In this report, we describe our approach to comprehensive outbreak suppression and report an initial case series of COVID-19 positive patients receiving home-based services. We implemented enhanced infection control procedures across our affiliates, and we communicated these protocols to our offices using multi-faceted methods. Using custom built software applications enabling us to track patient and employee cases and exposures, we leveraged current public health recommendations to identify cases and to suppress transmission. In the 100-day period between January 20, 2020 and April 30, 2020, our affiliates provided services to 67 COVID-19 positive patients (<0.3% of census). Twenty patients were referred to home health post hospitalization for COVID-19 related illness, whereas 47 were found to have COVID-19 while living in community settings. Of those who were found to have COVID-19 in the community, 17 (39%) required subsequent hospitalization. Hospitalized patients had an average age of 74.5 ± 18, and 53% were male. There were 13 deaths (76%) among those hospitalized from the community with COVID-19 related illness. A highly coordinated and frequently communicated approach to infection control, case identification and employee screening can be performed by home health and personal home care organizations. Studies that further assess risks and predictors of illness severity in home-based COVID-19 patients are needed.

## Background

The acute respiratory disease COVID-19, caused by the novel Coronavirus SARS-CoV-2, is a worldwide pandemic affecting millions of people. Since first being discovered in late 2019 in Wuhan, China,^1^ the virus has proliferated swiftly and has caused over 5 million infections and over 350,000 deaths.^2^ Studies have shown that older individuals with higher numbers of chronic medical conditions are at risk for being most severely ill from COVID-19.^3^ Medicare beneficiaries that have higher numbers of chronic conditions are the highest utilizers of home health services, with one-quarter of beneficiaries with six or more chronic conditions receiving 13 or more visits during the year.^4^ However, the manner in which COVID-19 is affecting home health and personal home care providers and patients has not yet been characterized. Our affiliates provide home health or personal home care services in 25 states. To address the threat posed by COVID-19, we developed a comprehensive outbreak preparedness and mitigation strategy, with a primary objective of protecting home health and personal home care patients. In this report, we present the mitigation methods we have utilized in our home health and personal home care affiliates in the 100 days since the first case of COVID-19 was confirmed in the U.S. on January 20, 2020. In addition, we report a COVID-19 positive case series of home health and personal home care patients, summarizing our initial experience in supporting patients during the pandemic.

## Methods

We brought together a multi-disciplinary team of medical, clinical, communications, operations, compliance, legal and risk management, as well as human resources leaders throughout our organization and formed an Outbreak Preparedness and Action Committee. The mission of the committee was to prepare for potential outbreaks and to act when necessary to protect, support, and serve patients and our employees. The committee developed a comprehensive preparedness plan and served as a means of consolidating internal and external communications regarding COVID-19 questions, planning, and response. Beginning in early February 2020, we began monitoring the global situation daily. Our principal monitoring source was the Johns Hopkins University Coronavirus Resource Center,^2^ as well as the Centers for Disease Control and Prevention (CDC) and World Health Organization’s (WHO) COVID-19 situation rooms.

When assessing individuals with a fever and lower respiratory symptoms, such as coughing or shortness of breath, or potential exposures, we utilized the CDC’s infection control guidance for healthcare professionals about Coronavirus.^5^ A COVID-19 case was defined as a positive nucleic acid test for SARS-CoV-2 RNA. We built a secure, cloud-based web application to enable capture of confirmed cases and exposures from all affiliate sites. The application leverages a QuickBase (QuickBase, Inc., Cambridge, MA) data structure to capture confirmed cases and potential exposures from sites across the U.S. Entry of new cases auto-notified our team of nurses, who then advised the operations team at our affiliate sites to assist with planning and triage of cases. The clinical and operational plan included reinforcement and training on necessary quarantine and isolation procedures, as well as ordering additional personal protective equipment (PPE) supply. Entry of new employee cases or exposures triggered an auto-notification to that location’s human resources partner, who then worked with the clinical team and the employee to support triage, isolation at home if needed, and eventual return to work. To optimize our ability to visualize COVID-19 positive patients and employees by site, we developed a business intelligence application, leveraging Power BI (Microsoft Corp, Redmond, WA). The leadership teams used the visualization application as a “real time situation room” that enabled us to deploy specific mitigation tactics as cases emerged.

**Figure 1.**
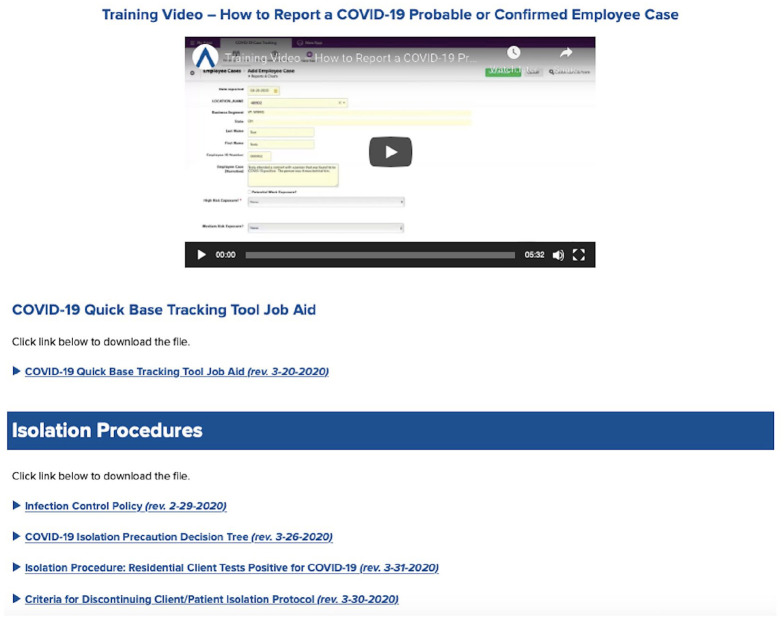
COVID-19 educational and practical resource hub.

Comprehensive training on infection control policies and procedures was deployed through a combination of intranet resources as well as on-site and web-based live meetings (Figure 1). The infection control measures were adapted from the U.S. Centers for Disease Control and Prevention,^5^ and the educational training enabling appropriate implementation of these measures was developed by our nursing quality team through a variety of live and recorded web meetings and slide presentations, videos, and written policy and instructional documents. In order to streamline the procurement and distribution of PPE to our home health and personal home care affiliates, we formed a new central supply function. PPE kits were assembled and shipped to all locations, in addition to allotments of hand sanitizer, cleaning materials, and other items required to effectuate optimal infection control. We also implemented additional cleaning and disinfection protocols in our offices. Leadership had daily management team conference calls and used a web based collaboration platform (Microsoft Teams, Microsoft Corp, Redmond, WA) to share daily operational documents and track PPE shipments to our branch locations.

**Figure 2.**
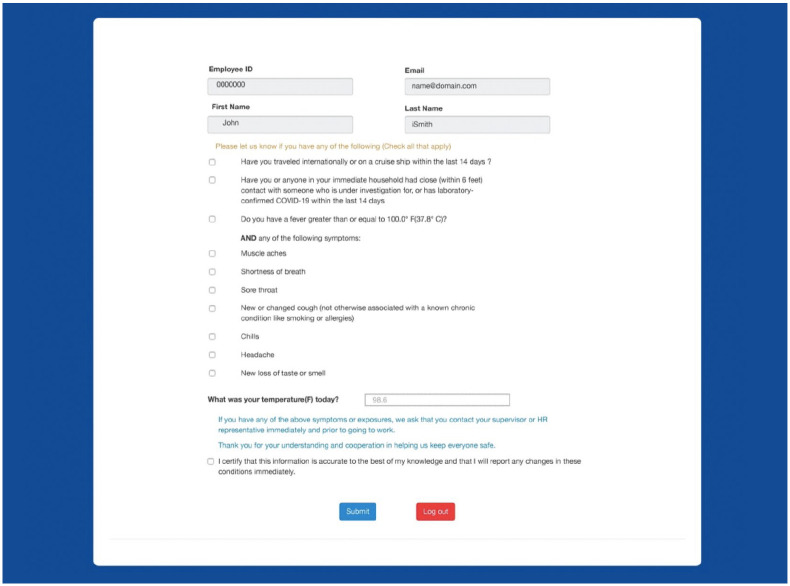
Employee symptom screening application.

To limit visitors to home care and home health offices as a potential vector of virus transmission, we enacted a policy that limited visits to all offices by people who are sick and posted signs near the entrance of our offices to remind sick visitors that they should not visit. In order to screen and prevent employees from coming to work sick, we developed a cloud-based symptom-screening application. For self-screening, all employees were asked to record their temperature daily and answer simple screening questions as shown (Figure 2). Symptomatic employees were isolated at home and tested for COVID-19 where testing was available. Where testing was not available, employees were prohibited from working until they met return to work requirements. For any COVID-19 positive employees, we isolated the employee at home until they met the CDC’s return to work criteria for healthcare workers.^6^

To enable employees across all locations to have access to the most current information, policies, and training materials, we developed and deployed over 100 COVID-19 outbreak prevention and action resource materials for employee use. This resource library was posted to our organizational intranet as well as our employee mobile app and updates were also communicated by email to the organization three times per week. The resource library is available here.

## Results

In the 100-day period between January 20, 2020 and April 30, 2020, our home health and personal home care affiliates provided services to 67 COVID-19 positive patients (<0.3% of census). 20 patients were referred to home health post hospitalization for COVID-19 related illness, whereas 47 were found to have COVID-19 while living in community settings. In 100% of cases, when a COVID-19 case was confirmed or suspected, caregivers wore personal protective equipment in the home and education of cohabitating individuals was performed. Of those who were found to have COVID-19 in the community, 17 (39%) required subsequent hospitalization. Hospitalized patients had an average age of 74.5 ± 18, and 53% were male. There were 13 deaths (76%) among those hospitalized from the community with COVID-19 related illness. Patients who died were approximately 5 years younger than those who did not.

## Discussion

We report 67 cases of COVID-19 in the first 100 days of the U.S. COVID-19 outbreak in a home health and personal home care population. At a prevalence of census of less than one-half of 1%, this infection prevalence rate is considerably lower than rates reported in congregate care settings.^7^ In our early COVID-19 experience, 30% of cases were referred to home health after a hospitalization during which the patient was found to have COVID-19. About 70% were found to have COVID-19 in community settings, and of those 39% required hospitalization. Among those requiring hospitalization, three-quarters died. Older individuals with higher numbers of chronic medical conditions have been shown to be at risk for being most severely ill with COVID-19,^3^ however in our affiliates’ early experience, those who died were approximately 5 years younger than those who did not. Further evaluation of additional risks that may be associated with increased likelihood of hospitalization and death in a community-based home care population, including number and type of preexisting medical conditions and social determinants of health, are needed.

While the overall prevalence of COVID-19 among our affiliates to date is low, our organization is taking considerable steps to continue to identify and mitigate potential cases and exposures to keep infection rates low. Employee symptom and temperature screenings, and the use of appropriate PPE will continue to be critical components of outbreak mitigation for all patient-facing caregivers and clinicians. In addition, regular workforce surveillance testing is likely to play an increasingly important role in those visiting patients in home settings. Serological assays to detect SARS-CoV-2 antibodies are rapidly becoming available and will be critical to estimate the prevalence of infections, including those who are asymptomatic.^8^ Following infection, detectable IgM and IgG antibodies develop within days to weeks of symptom onset in most infected individuals.^9-11^ While it is presently premature to use such assays to determine whether individuals are immune to reinfection, there may be greater current value in utilizing an antibody test’s negative predictive value. Initially, we have begun utilizing point of care (POC) antibody testing for clinicians and caregivers who visit congregate care settings. While any symptomatic or COVID-19 positive employee continues to be disallowed from working until they meet return to work guidelines, we are also beginning to screen asymptomatic visiting clinicians and caregivers using a rapid POC test for IgM and IgG antibodies against SARS-CoV-2. While antibody tests are not appropriate for diagnosing symptomatic individuals, we believe that using antibody tests that have adequate negative predictive value to routinely screen asymptomatic home care workers, particularly those who visit congregate care settings, may be helpful. A negative result on such a COVID-19 POC antibody test performed on home health clinicians and caregivers, in addition to daily documentation of a lack of symptoms or fever, can provide additional reassurance to patients, family members and leaders of congregate care settings that visiting caregivers and clinicians have a low likelihood of transmitting infection to patients and residents.

A highly coordinated and frequently communicated approach to infection control, case identification and caregiver and clinician screening can be performed by home health and personal home care organizations. Such methodology is especially important in a pandemic such as COVID-19, where the home is emerging to be a preferred place for many affected patients to isolate and recover. Studies that further evaluate medical and social determinant risks and evaluate predictors of severity of illness in home-based COVID-19 patients are needed.
